# Exploring the impact of urban expansion on urban green land use efficiency: a case study of Chengdu-Chongqing urban agglomeration

**DOI:** 10.3389/fpubh.2025.1596250

**Published:** 2025-09-25

**Authors:** Lanyue Zhang, Cuiping Zhang, Chen Gao, Chen Wang

**Affiliations:** ^1^School of Digital Economics, Sichuan University Jinjiang College, Meishan, Sichuan, China; ^2^Department of Economics and Management, Weifang Engineering Vocational College, Qingzhou, China; ^3^School of Finance, Hunan University of Technology and Business, Changsha, China; ^4^School of Economics, Beijing Technology and Business University, Beijing, China

**Keywords:** urban expansion, urban green land use efficiency, ESDA, GTWR, spatial–temporal heterogeneity

## Abstract

**Introduction:**

Efficient utilization of limited land resources to maximize economic, social, and ecological benefits is critical for enhancing urban green land use efficiency and achieving sustainable urban development.

**Methods:**

This research integrates multi-source socioeconomic data to analyze 16 cities within the Chengdu-Chongqing urban agglomeration from 2006 to 2022. Employing exploratory spatial data analysis, standard deviation ellipse modeling, bidirectional fixed-effects regression, and geographically and temporally weighted regression, we examine the spatial-temporal evolution of urban green land use efficiency and the impact of urban expansion on urban green land use efficiency.

**Results:**

The result shows that: (1) urban green land use efficiency exhibits a significant upward trend, reflecting a core-periphery structural evolution. (2) The spatial spillover effect of urban green land use efficiency remains consistently negative, particularly pronounced in the core cities of Chengdu and Chongqing. (3) Urban expansion negatively affects urban green land use efficiency, with a coefficient estimate of −0.185 in the fixed-effects model, and the GTWR model further reveals a deepening adverse impact from 2006 to 2022.

**Discussion:**

These findings underscore the necessity of adopting “smart growth” strategies and provide policymakers with evidence-based insights for optimizing urban spatial planning and intercity collaboration.

## 1 Introduction

The accelerated productivity advancement since the 21st century has driven unprecedented urban expansion, fundamentally transforming land use patterns globally (1–3). While rapid urbanization has facilitated socioeconomic development, unregulated population concentration and escalating land demands (4, 5) have intensified conflicts with finite land resources, leading to extensive land utilization and ecological degradation (6–8). China’s urbanization trajectory exemplifies these challenges. With urbanization rates reaching 66.16% by 2023 (exceeding global averages by 10%), the Chengdu-Chongqing urban agglomeration exemplifies a paradigmatic case of low-density spatial expansion (9). As China’s sole inland dual-core urban agglomeration with a permanent population exceeding 100 million, its development is propelled by the convergence of multiple national strategies. The 2016 Chengdu-Chongqing Urban Agglomeration Development Plan institutionalized a “dual-core drive, multi-polar support” spatial framework, which was further elevated in 2020 through the national Twin-City Economic Circle strategy. This policy framework facilitated specialized industrial clustering under the “one city, multiple parks” model—Chongqing prioritized intelligent connected vehicles and integrated circuit manufacturing. At the same time, Chengdu focused on digital economy and biology R&D. Although this approach accelerates high-tech industry agglomeration and industrial upgrading, it concurrently exacerbates ecological space encroachment, destabilizing the land-green-urban-ecology (LGUE) equilibrium (10–13).

The ecological governance of the Chengdu-Chongqing urban agglomeration remains predominantly reliant on post-event restoration measures, with a lack of a stringent regional constraint mechanism that spans the entire area. This research examines the Chengdu-Chongqing urban agglomeration, a representative emerging economy in China, to scientifically assess the spatial–temporal impacts of urban expansion on urban green land use efficiency (UGLUE). The research holds dual significance: theoretically, it reveals the regulatory mechanisms of policy tool combinations on urban land systems; practically, it provides an institutional innovation model for latecomer urban agglomerations worldwide. The findings contribute to maintaining the economic, social, and ecological benefits of urban land use while addressing the core challenge of SDG 11 (“Sustainable Cities and Communities”)—balancing human settlement needs with the prevention of predatory land ecosystem development (14, 15).

Current research on UGLUE has evolved along three primary trajectories: theoretical conceptualization, policy formulation, and methodological innovation in indicator development and measurement. Notably, multidimensional indicators have become standard practice, with the “economy-resource-environment” synergy framework dominating contemporary analyses (16, 17), effectively quantifying green performance. Relevant studies show that UGLUE has firm spatial heterogeneity (3, 18). Much research has focused on the spatial heterogeneity of UGLUE indicators. For instance, resource-based cities require adjustments to the index weights to account for variations in resource endowment and policy orientation (3). Similarly, for urban agglomerations in China, it is crucial to consider internal developmental disparities when selecting appropriate indicators (17). On the other hand, to achieve the goals of urban “smart growth” and green transformation, the academic community has extensively discussed the driving mechanisms behind UGLUE. Policy regulation, technological innovation, social and economic structure, and urban spatial form are the key driving factors influencing LGUE (4, 16, 19–22). For example, Dong et al. (23) used geographic detectors to identify the driving factors of UGLUE, revealing a significant interaction between human and environmental factors. Specifically, the q value between vegetation and building height was 0.298. Liu et al. (24) examined the impact of growth target management and regional competition on UGLUE using a spatial–temporal gravity spatial weight matrix and a spatial self-hysteresis model. They found that both factors had a positive impact on land use efficiency, and heterogeneity analysis indicated that this effect was more pronounced in eastern cities and urban agglomerations. Li et al. (19) applied the GTWR model to explore the driving mechanisms of UGLUE and found that the influence of technological factors and pollution emissions was increasing.

In addition to its spatial characteristics and driving mechanisms, the benefits of UGLUE have also attracted increasing scholarly attention. Recent studies have evaluated the ecological services provided by various types of urban green spaces—such as parks, community greenery, and rooftop vegetation—and quantified their economic value to offer actionable insights for urban policymakers (25). UGLUE plays a critical role in mitigating the urban heat island effect. Well-distributed and adequately sized green spaces can reduce surface and ambient temperatures through evapotranspiration, serving as “green air conditioners” during heat waves, thereby improving thermal comfort and reducing energy demand. Beyond ecological advantages, UGLUE also generates substantial socioeconomic benefits (26, 27). Empirical evidence shows that access to green spaces enhances residents’ well-being and life satisfaction by relieving stress, restoring attention, enriching esthetic experience, and promoting physical activity (28). Moreover, efficient green land use stimulates the growth of green industries such as eco-tourism, landscape horticulture, and urban ecological agriculture, generating new employment opportunities and contributing to sustainable economic development (29).

Urban expansion represents a dynamic process in which urban space sprawls outward from the core to the periphery, encompassing changes in land use, population distribution, and infrastructure deployment. It is regarded as a central issue in urban planning and governance (30). Current approaches to measuring urban expansion include: (1) land cover change analysis, which employs multi-temporal remote sensing imagery and GIS tools to quantify the growth of built-up areas (e.g., residential, commercial, and industrial land) and identify shifts in spatial patterns (31, 32); (2) morphological metrics, such as compactness, fractal dimension, and landscape fragmentation, which assess the geometric characteristics of urban sprawl (33, 34); and (3) population-based measures, where shifts in population density are used to evaluate urban expansion efficiency (35, 36). Notably, Sustainable Development Goal 11.3.1 defines the Land Consumption Rate to Population Growth Rate (LCRPGR) ratio, which captures the alignment between spatial growth and demographic trends—thereby offering an integrated perspective on whether expansion is occurring in a coordinated or unregulated manner, and whether it poses risks to valuable resources such as farmland and urban green space.

Urban expansion is frequently associated with adverse environmental consequences (37). One of the most immediate impacts is the large-scale conversion of agricultural land, forests, and wetlands, leading to a significant reduction in urban ecosystem services, biodiversity loss, and weakened carbon sequestration capacity (38). In addition, the proliferation of residential and commercial buildings results in substantial energy consumption and elevated carbon emissions. Industrial zone expansion—another significant facet of urban growth—often concentrates pollution and carbon-intensive activities within specific zones. Furthermore, unregulated expansion can lead to urban congestion and infrastructure redundancy (39), primarily due to sprawling urban forms that lengthen commuting distances and intensify transportation pressure.

Existing literature demonstrates that urban expansion exerts dual effects on urban land use efficiency through three primary mechanisms: scale effects, technological diffusion, and institutional factors. From a scale economy perspective, factor concentration resulting from urban expansion enhances marginal output through optimized resource allocation intensity. Zhou et al. (40) empirically confirmed this positive correlation between economic density and green efficiency in their study of Chengdu-Chongqing’s “dual-core-driven” growth model, which promotes industrial chain integration and reduces per-unit land energy consumption. This agglomeration effect further facilitates technological diffusion (41), as evidenced by the broader adoption of intelligent transportation systems and green building technologies in new urban districts. Such technological upgrades face higher implementation barriers in established urban areas due to multi-stakeholder coordination challenges and elevated investment costs, whereas emerging districts can directly implement cutting-edge solutions to minimize land waste and pollution (42). In terms of urban density, disorderly spatial expansion fails to account for economic restructuring trends and population distribution. This mismatch between urban growth and demographic shifts may lead to “ghost towns” and underutilized infrastructure, reducing public service efficiency and increasing energy consumption per unit area (43, 44). Moreover, Expansion policies without strict regulatory oversight exacerbate the adverse effect. For example, in the Chengdu-Chongqing urban agglomeration, the overlap between industrial land and ecological protection zones is substantially higher than in comparable regions. This undermines the ability of high-tech industries’ green spillover effects to compensate for environmental degradation.

The literature review reveals that a multidimensional research framework has been established to examine the complex relationship between urban expansion and UGLUE, with significant progress in both theoretical development and methodological innovation. Scholars have increasingly adopted integrated analytical frameworks that account for economic, social, and environmental dimensions to uncover the spatial heterogeneity and driving mechanisms of UGLUE. However, several research gaps remain. First, relatively few studies have provided empirical evidence on the impact of urban expansion on UGLUE. Given that both urban expansion and green land use efficiency are core issues in urban planning and management, identifying the causal relationship between the two is of critical importance. Second, while urban expansion is inherently spatially heterogeneous, its effects on UGLUE are also spatiotemporally variant—yet existing studies rarely examine this dynamic heterogeneity. Moreover, research focusing on rapidly urbanizing regions in emerging economies remains limited, particularly in the context of the Chengdu–Chongqing urban agglomeration, where large-scale urban expansion is actively underway but lacks systematic empirical analysis.

In addition, many existing spatial analytical methods remain fragmented or confined to single-scale approaches, failing to capture the dynamic evolution of efficiency-related spatial clustering. Traditional studies on driving mechanisms often rely on global regression models, which are inadequate for revealing the nuanced spatiotemporal variation in the effects of urban expansion.

**Figure 1:**
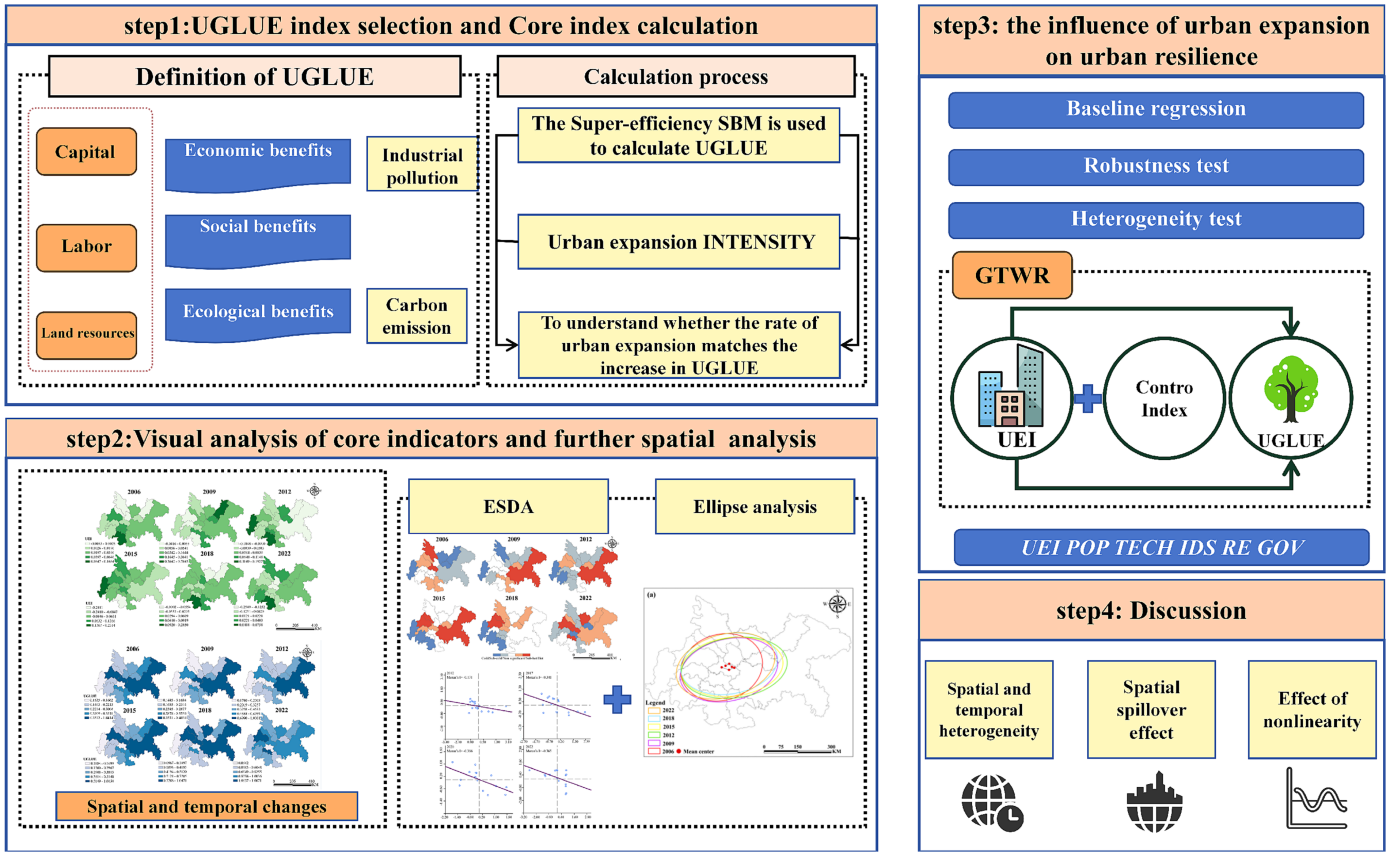
Research framework.

This study addresses a critical scientific question: whether urban expansion affects UGLUE, and if so, in what direction. To this end, the research makes four key contributions that extend the existing literature: (1) Causal Identification: This study provides empirical evidence confirming the impact of urban expansion on UGLUE. To address potential endogeneity, an instrumental variable (IV) approach is employed to identify the causal relationship between the two robustly. (2) Contextual Innovation: The Chengdu–Chongqing urban agglomeration, a representative case of a rapidly urbanizing region in an emerging economy, is selected as the study area. Compared with other major urban clusters in China, this region has undergone relatively late-stage development and ecological governance, but possesses considerable potential for economic restructuring. Over the past decade, its urbanization rate has exceeded the national average by more than 50%. As it transitions from scale-driven expansion to quality-oriented growth, the region offers a unique empirical context to explore the dynamic interplay between urban growth and land-use efficiency. (3) Multiscale Spatial Analysis: A comprehensive spatial analytical framework is constructed. Moran’s I is used to detect spatial autocorrelation of UGLUE, while hot spot analysis captures spatial gradients in efficiency evolution. In addition, the incorporation of standard deviation ellipse modeling allows for the identification of directional shifts and spatial convergence patterns, overcoming the limitations of one-dimensional spatial analysis commonly used in previous studies. (4) Spatiotemporal Modeling: Building upon traditional heterogeneity and robustness tests, this study employs a Geographically and Temporally Weighted Regression (GTWR) model to construct a spatiotemporal heterogeneity framework. The model effectively captures the dynamic effects of urban expansion on UGLUE across both time and space, offering precise spatial coordinates to support targeted policy interventions (Figure 1).

## 2 Study area

The Chengdu–Chongqing urban agglomeration (also known as the Chengdu–Chongqing Twin-City Economic Circle) is a nationally designated urban cluster, comprising 27 districts and counties in Chongqing (including parts of Kaizhou District and Yunyang County), as well as 15 cities in Sichuan Province, including Chengdu, Zigong, and others. According to the Chengdu–Chongqing Twin-City Economic Circle Integration Development Index Report (2022–2023), the region has experienced rapid socioeconomic growth. In 2022, its GDP reached 7.8 trillion Yuan, accounting for 6.4% of China’s national total, with a per capita GDP of 79,000 Yuan.

In terms of urbanization, cities such as Mianyang, Zigong, Nanchong, Yibin, and Wanzhou each have urban populations exceeding one million. Notably, Mianyang and Yibin reported total GDPs exceeding 300 billion Yuan. The combined GDP of cities other than the core areas of Chengdu and central Chongqing reached 4.541 trillion Yuan, representing 58.5% of the entire agglomeration’s economic output.

From an ecological perspective, joint environmental protection efforts have yielded significant results. The number of days with good air quality in Chongqing and Sichuan increased from 300 and 287 days in 2016 to 332 and 326 days in 2022, respectively, with compliance rates of 90.96 and 89.3%.

**Figure 2:**
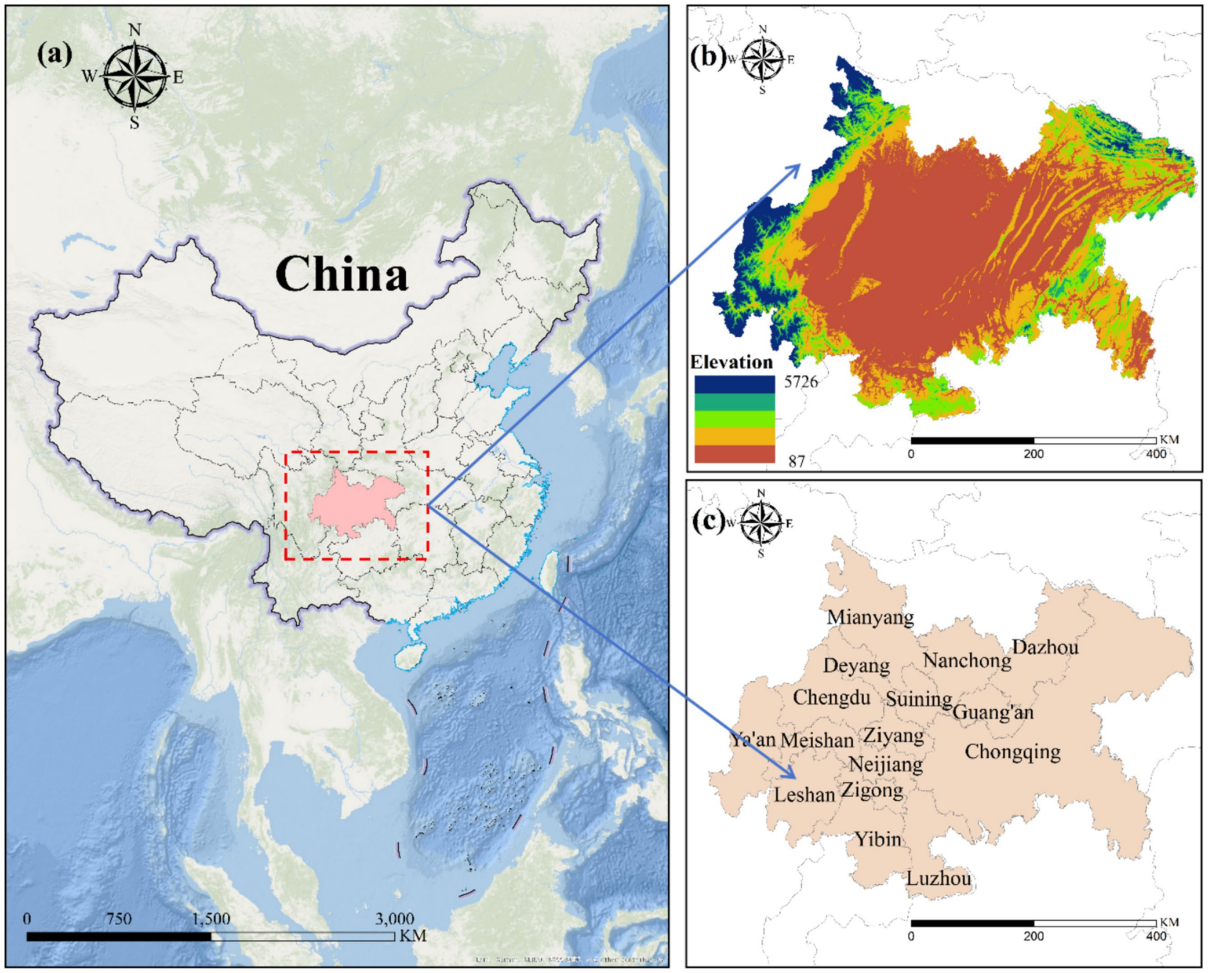
Location map of the Chengdu-Chongqing urban agglomeration. **(a)** A geographical map of China’s location; **(b)** DEM images for the Chengdu-Chongqing urban agglomeration; **(c)** for the sample of cities in the Chengdu-Chongqing urban agglomeration.

However, the rising demand for sustainable development has rendered traditional rapid expansion models obsolete, prompting a necessary restructuring of the urban system. This study focuses on 16 representative cities, including Chengdu, Chongqing, Mianyang, and Deyang (as shown in Figure 2), which differ considerably in terms of economic and social development. The region’s basin and hilly topography pose constraints on transportation networks, industrial layout, and ecological conservation, while also complicating responses to natural disasters and environmental degradation. Against this backdrop, the present research examines the development challenges faced by the Chengdu–Chongqing urban agglomeration and investigates the impact of urban expansion on urban resilience.

## 3 Index system and methodology

### 3.1 Index system

The evaluation of UGLUE typically examines input factors, expected outputs, and undesired outputs. Conventional input factors include capital investment, land utilization, labor input, and environmental governance expenditures, while output indicators cover economic benefits like industrial value-added, social benefits such as employment rates, and environmental benefits including green space coverage. Undesired outputs mainly consist of environmental pollutants such as air emissions, wastewater discharge, soil contaminants, and excessive energy consumption. Drawing upon established literature, we have selected and standardized these indicators as detailed in Table 1 (2, 3, 45–47). For the core explanatory variable, this study employs UEI, calculated using annual changes in built-up area. Regarding control variables, five key factors are incorporated: (1) population density (POP), measured as persons per square kilometer of built-up area, influences UGLUE through resource utilization intensity; (2) Resource efficiency (RE), quantified by industrial solid waste utilization rate, may reduce waste generation; (3) Technological capacity (TECH), represented by patent grants, reflects potential for clean energy adoption; (4) Industrial structure (IDS), measured as tertiary sector value-added to GDP ratio, affects green industry distribution; and (5) Government intervention (GOV), indicated by fiscal expenditure-to-GDP ratio, reflects policy strictness.

**Table 1:** UGLUE index system.

Criterion layer	Feature layer	Indicator layer
Input	Capital factor input	Investment in fixed assets (10,000 Yuan)
	Land factor input	Built-up area (km^2^)
	Labor factor input	Number of Employees in Secondary and Tertiary Industries
	Environment factor input	Environmental input (10,000 Yuan)
Expected output	Economic benefits	value of the secondary and tertiary industries
	Social benefits	General Public Budget Revenue (10,000 Yuan)
		Savings balance of residents (10,000 Yuan)
	Ecological benefits	Per capital green area (person/m^2^)
Undesired output	Industrial pollution output	Industrial wastewater discharge (10,000 tons)
		Industrial sulfur dioxide emissions (10,000 tons) Industrial nitrogen oxide emissions (10,000 tons) Industrial particulate matter emissions (10,000 tons)
	Carbon emission output	Carbon emissions (tons)

The data used in this study were primarily obtained from the China City Statistical Yearbook, China Environmental Statistical Yearbook, and China Science and Technology Statistical Yearbook, as well as from the official website of the National Bureau of Statistics. These sources provided annual indicators such as built-up area, fiscal expenditure, industrial structure, and population density at the city level. In addition, data on technological capacity—such as the number of granted patents—were retrieved from the China National Intellectual Property Administration (CNIPA). Indicators related to resource use efficiency were derived from publicly available reports issued by provincial and municipal ecological and environmental departments, including the China Environmental Status Bulletin. Built-up area data were further validated and supplemented using remote sensing imagery and annual statistical integration.

The study period spans 2006–2022, there is no missing data in the relevant data, and all the data in this study were standardized, so there was no influence of dimensional differences or extreme values.

### 3.2 Methodology

#### 3.2.1 Core variable measure

##### 3.2.1.1 Urban green land use efficiency

This research utilizes the super-efficient SBM model, developed by Tone, to assess UGLUE. The super-efficient SBM model is a non-radial efficiency measurement method within the framework of Data Envelopment Analysis (DEA). Its key advantages include the ability to handle non-expected outputs and provide more comparable efficiency values (48). The specific Equations 1 and 2 are as follows:


(1) $$\min p=\frac{\frac{1}{m}\sum_{i=1}^{N}(\frac{\bar{x}}{x_{\mathrm{ik}}})}{\frac{1}{s_{1}+s_{2}}(\sum_{r=1}^{s_{1}}\frac{\bar{y^{d}}}{y_{\mathrm{rk}}^{d}}+\sum_{q=1}^{s_{2}}\frac{\bar{y^{b}}}{y_{\mathrm{qk}}^{b}})}$$

(2) $$s.t.\{\begin{array}{l} \bar{x}\geq \sum_{j=1,\neq k}^{N}x_{\mathrm{ij}}\lambda_{j};\bar{y^{d}}\leq \sum_{j=1,\neq k}^{N}y_{\mathrm{rj}}^{d}\lambda_{j};\bar{y^{d}}\geq \sum_{j=1,\neq k}^{N}y_{\mathrm{qj}}^{d}\lambda_{j}\\ \bar{x}\geq x_{k};\bar{y^{d}}\leq y_{k}^{d};\bar{\;y^{b}}\geq y_{k}^{b}\\ \lambda_{j}\geq 0;i=1,2,\cdots ,m;j=1,2,\cdots ,N\\ r=1,2,\cdots s_{1};q=1,2\cdots ,s_{2} \end{array}$$

Where, p represents the relative UGLUE value of DMUs; I, r, q are input factors, desired output, and undesired output, respectively; m, $s_{1}$ , $s_{2}$ represent the types of input factors, expected outputs and non-expected outputs; x, $y^{d}$ , $y^{b}$ , are the input matrix, the expected output matrix, and the unexpected output matrix, respectively; $\bar{x}$ ; $\bar{y^{d}}$ ; $\bar{y^{b}}$ are the slack variable of input, desired output, and undesired output, respectively, λ is the weight coefficient.

##### 3.2.1.2 Urban expansion intensity

This study comprehensively considers various quantitative methods for urban expansion, including the expansion rate, intensity index, elasticity coefficient, morphological index, center of gravity migration model, and land use change intensity index. Based on the requirements of spatial–temporal analysis, the UEI index is selected as the core explanatory variable. This index is used to quantitatively characterize the dynamic characteristics of land development in the target area over a specific time period. The specific Equation 3 is as follows:


(3) $${\mathrm{UEI}}_{i}=\frac{\mathrm{UL}A_{i}^{t_{2}}-\mathrm{UL}A_{i}^{t_{1}}}{\mathrm{TL}A_{i}\times n}\times 100\%$$

Where the UEI index of city i $\mathrm{TL}A_{i}$ is the total built-up area of city i (km^2^); n is the research time span. In order to better understand the temporal changes against the background of the high-speed expansion of the Chengdu-Chongqing urban agglomeration, the UEI index in this research is annually calculated, and n is 1. The area of the city is in year *t2* and year *t1*.

##### 3.2.1.3 Urban sprawl index

To verify the robustness of our findings regarding urban expansion’s impact on UGLUE, we conducted supplementary regression analyses using the urban sprawl index as an alternative to UEI. The urban sprawl index quantifies disordered spatial development patterns, characterized by low-density decentralization, single land function, and inefficient distribution of population and economic activities. Such sprawl typically generates negative externalities including ecological degradation, traffic congestion, and resource misallocation. Following the methodology of Lv et al. (49), we computed annual sprawl indices for the Chengdu-Chongqing urban agglomeration (2006–2022) by integrating nighttime light intensity and population density raster data (calculation formula omitted for brevity).

#### 3.2.2 Exploratory spatial data analysis

##### 3.2.2.1 Moran’s I

This study used Moran’s I index for spatial auto-correlation analysis. As a core tool in geo-spatial statistics, this method effectively addresses the issue of spatial heterogeneity identification that traditional statistical methods struggle with, by quantifying the degree of spatial dependence in unit values. It also overcomes technical challenges in detecting agglomeration patterns and assessing spatial spillover effects (50), incorporating both the global and local Moran indices. The global Moran index, in particular, is a key indicator used to determine whether the research objects exhibit spatial agglomeration properties. The specific Equation 4 is as follows:


(4) $$I=\frac{n\sum_{i=1}^{n}\sum_{j=1}^{n}W_{\mathrm{ij}}(y_{i}-\bar{y})(y_{j}-\bar{y})}{n\sum_{i=1}^{n}\sum_{j=1}^{n}W_{\mathrm{ij}}\sum_{i=1}^{n}{\left(y_{i}-\bar{y}\right)}^{2}}$$

Where *n* is the number of samples, the research unit is 16 prefecture-level cities in the Chengdu-Chongqing urban agglomeration, so 16 is selected. $y_{i}$ $y_{j}$ represents the observed values of cities i and j, $\bar{y}$ is the mean value of UGLUE, and w represents the spatial weight.

The local Moran index is used to describe the numerical similarity between a research unit and its neighboring units; it is a specific indicator of spatial agglomeration characteristics. Its measurement (Equation 5) is as follows:


(5) $$I_{i}=\frac{\left(y_{i}-\bar{y}\right)}{\sum_{i=1}^{n}{\left(y_{i}-\bar{y}\right)}^{2}}\sum_{j=1}^{n}W_{\mathrm{ij}}(y_{i}-\bar{y})$$

##### 3.2.2.2 Hotspot analysis

According to the first law of geography, everything is correlated, and the green use efficiency of urban land reflects this principle. Cold and hot spot analysis can effectively identify areas with spatial agglomeration effects, primarily using the Getis-Ord Gi* index, as shown in Equations 6 and 7:


(6) $$G_{i}^{*}{\left(d\right)}^{2}=\sum_{j=1}^{n}W_{\mathrm{ij}}(d)X/\sum_{j=1}^{n}X_{\mathrm{ij}}$$

(7) $$Z{\left(G_{i}^{*}\right)}^{2}=G_{i}^{*}-E(G_{i}^{*})/\sqrt{\mathrm{Var}(G_{i}^{*})}$$

##### 3.2.2.3 Standard deviation ellipse model

The standard deviation ellipse model is a key method in spatial analysis, used to describe the distribution patterns of spatial points and to quantify their directionality, expansibility, and agglomeration characteristics. This research employs the standard deviation ellipse model to analyze the spatial–temporal evolution of urban resilience and its subsystems in the Chengdu-Chongqing urban agglomeration under rapid urbanization over the past decade. For brevity, the detailed calculation process is omitted.

##### 3.2.2.4 GTWR

This research employs the GTWR model to establish an analytical framework for understanding the driving mechanism of urban expansion on land green use efficiency. By integrating spatial heterogeneity and temporal dynamics into the traditional Geographical Weighted Regression (GWR) framework, the model enables the simultaneous capture of spatiotemporal non-stationarity during parameter estimation, significantly enhancing the accuracy of analyzing the complex interaction effects within the land use system. Therefore, it is particularly suitable for revealing the intricate interactions within land use systems during rapid urbanization and for analyzing the spatial and temporal heterogeneity of land development intensity on land green use efficiency (51) as shown in Equation 8.


(8) $$y_{i}=\beta_{0}(u_{i},v_{i},t_{i})+\beta_{k}(u_{i},v_{i},t_{i})x_{\mathrm{ik}}+\varepsilon_{i},i=1,2,\ldots ,n$$

Where the observed value is the coordinate data of the Ith research object, the regression coefficient of the Ith research object, the Kth regression coefficient of the Ith research object, the value of the independent variable of the Ith research object, and the independent random error term.

## 4 Results

### 4.1 Spatial–temporal result analysis

#### 4.1.1 Urban expansion

**Figure 3:**
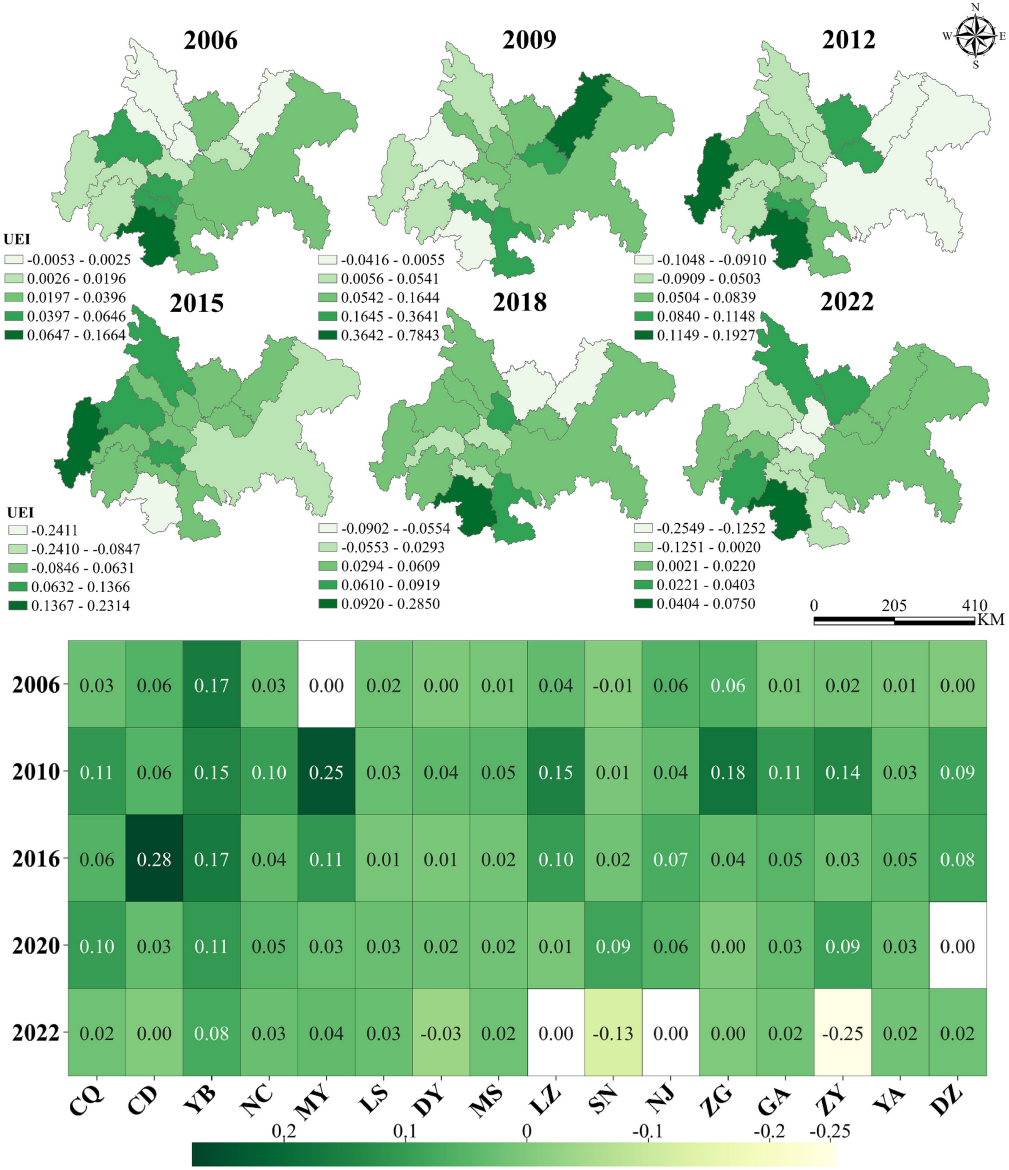
Spatial–temporal changes of UEI during 2006–2022.

This study employed ArcGIS Pro 3.0.2 to generate spatiotemporal evolution maps. Figure 3 indicates that the UEI in the Chengdu-Chongqing urban agglomeration demonstrated a distinct evolutionary trajectory from 2006 to 2022, characterized by three discernible phases. The initial rapid expansion phase (2006–2009) witnessed UEI values surging from 0.032 to 0.151, primarily driven by substantial infrastructure investments under national economic stimulus plans. This was followed by an adjustment phase (2010–2015) where UEI retreated to 0.032, reflecting the implementation of macroeconomic regulatory policies. The most recent fluctuation phase (2016–2022) exhibited an “M-shaped” pattern, peaking at 0.070 during 2016–2017 before declining to −0.008 by 2022, indicative of a strategic shift toward quality-driven “smart growth” paradigms.

Spatial analysis across the 16 cities reveals significant differentiation in UEI patterns. Yibin City emerged as the highest performer with an average UEI of 0.115, reaching a peak of 0.285 in 2018, attributable to its successful “industry-city integration” strategy that fostered clusters in the power battery and photovoltaic industries, generating over 200,000 new jobs. The dual-core cities of Chengdu (0.066) and Chongqing (0.047) maintained stable growth patterns, supported by strategic development zones such as Chongqing’s Liangjiang New Area and Chengdu’s Tianfu New Area. In contrast, cities like Leshan (0.033) and Suining (0.037) recorded lower UEI values, with Leshan constrained by its tourism-based economy and ecological protection requirements, while Suining faced challenges from delayed industrial transformation and weaker regional economic vitality. This spatial pattern clearly demonstrates that core node cities, including Chengdu, Deyang, Meishan, Ziyang, Yibin, and Chongqing, generally exhibited higher UEI values compared to peripheral cities with more limited land use demands.

#### 4.1.2 Urban green land use efficiency

**Figure 4:**
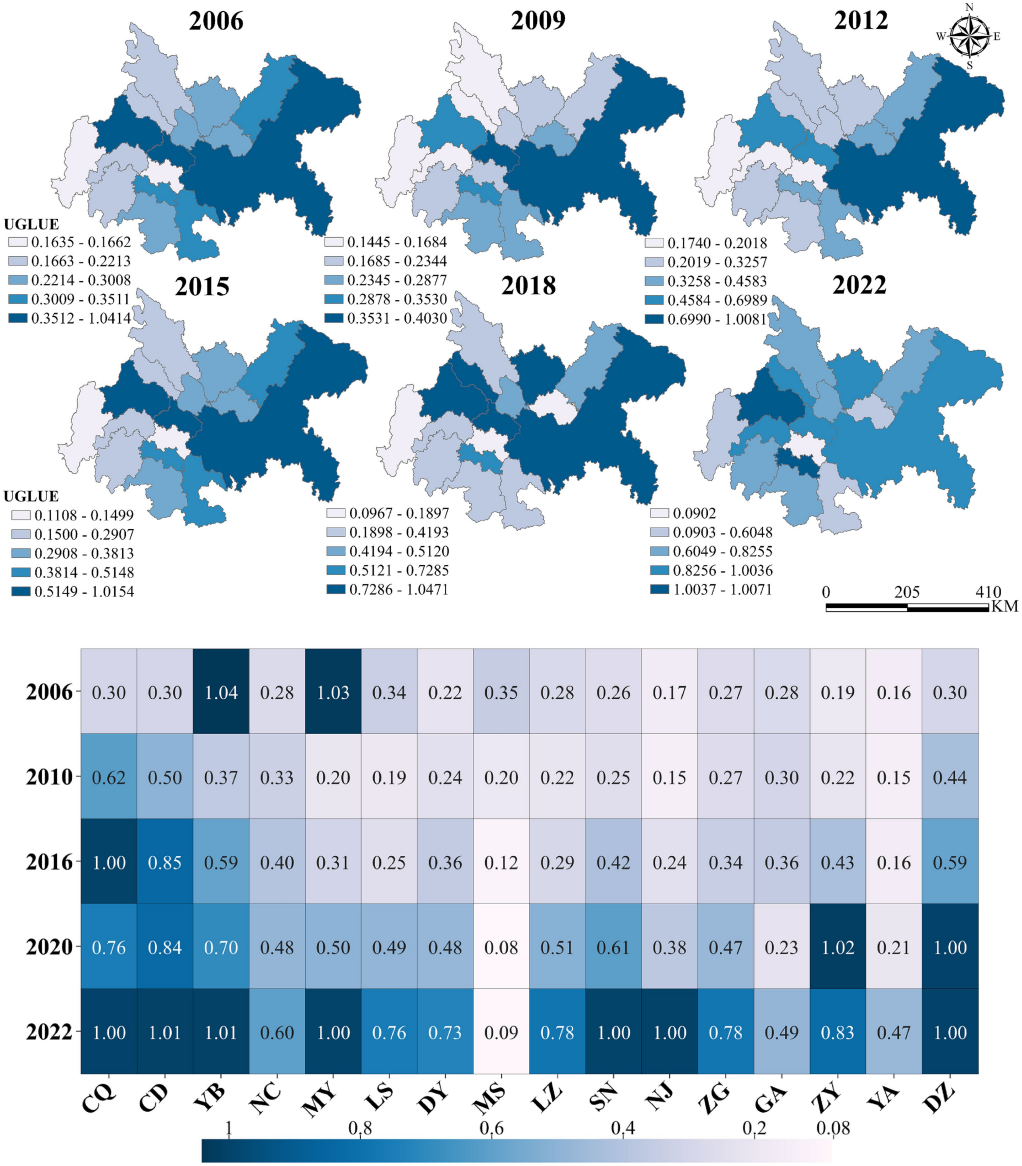
Spatial–temporal changes of UGLUE during 2006–2022.

This study utilized MATLAB R2024a to calculate the UGLUE. The UGLUE in the Chengdu-Chongqing urban agglomeration exhibited a dynamic three-phase evolution from 2006 to 2022 (Figure 4). The initial decline phase (2006–2010) saw UGLUE decrease by 19.70%, from 0.360 to 0.289, reflecting the region’s prioritization of economic growth over ecological considerations during rapid urbanization. This was followed by a slow recovery phase (2011–2015), where UGLUE gradually increased at an annual rate of 3.5%, reaching 0.439 by 2015, attributable to the implementation of ecological protection policies, particularly Chengdu’s pioneering establishment of environmental protection red lines and resource utilization limits.

The most significant transformation occurred during the rapid growth phase (2016–2022), with UGLUE surging 87.25% to 0.784, driven by comprehensive green development policies. Key initiatives included Chongqing Liangjiang New Area’s industrial access negative list, which reduced high energy-consuming land use, and large-scale clean energy projects like the Yibin Power Battery Industrial Park, following China’s carbon peak commitments. Spatial analysis reveals Chongqing (0.747) and Chengdu (0.732) as UGLUE leaders, benefiting from their dual-core status and extensive environmental regulations, while Meishan (0.276), Ya’an (0.194), and Neijiang (0.172) lagged due to weaker industrial bases and less stringent environmental controls. This spatial–temporal pattern demonstrates how policy interventions and industrial transformation collectively shaped the region’s green development trajectory.

### 4.2 ESDA and standard deviation ellipse analysis of UGLUE

#### 4.2.1 Moran’s I

This study is based on STATA 16.0 to use Moran’s I to analyze the UGLUE spatial autocorrelation characteristics of the Chengdu-Chongqing urban agglomeration from 2006 to 2022. As shown in Table 2, the global Moran’s I increased from −0.053 in 2006 to −0.367 in 2022, revealing that the UGLUE in the Chengdu-Chongqing urban agglomeration has consistently demonstrated negative spatial spillover effects, suggesting an inability of higher-UGLUE cities to effectively drive synchronous improvements in neighboring areas. The observed phenomenon may be attributed to an exacerbated “Siphon effect” within certain cities of the Chengdu-Chongqing urban agglomeration. For instance, Chengdu and Chongqing, as the two principal urban centers, have demonstrably amassed a substantial proportion of high-quality resources, particularly in human capital, technological innovation, and industrial structure. This concentration consequently leads to peripheral cities experiencing significant challenges, including the outward migration of skilled labor, inadequate financial resources, and limited access to advanced technologies. Consequently, the rate of UGLUE promotion is impeded. Furthermore, the preferential position of core cities in the process of industrial gradient transfer, characterized by the relocation of numerous pollution-intensive industries, exacerbates the non-green characteristics of land use in adjacent urban areas.

**Table 2:** Results of global Moran’s I.

Year	Moran’s I	E(I)	sd(I)	z	Year	Moran’s I	E(I)	sd(I)	z
2006	−0.053	−0.066	0.1751	0.111	2016	−0.273	−0.066	0.198	−1.000
2010	−0.247	−0.066	0.197	−0.904	2020	−0.386^*^	−0.066	0.208	−1.498
2014	−0.367^**^	−0.066	0.184	−1.624	2022	−0.365^*^	−0.066	0.200	−1.433

^***^p < 0.01, ^**^p < 0.05, ^*^p < 0.1.

**Figure 5:**
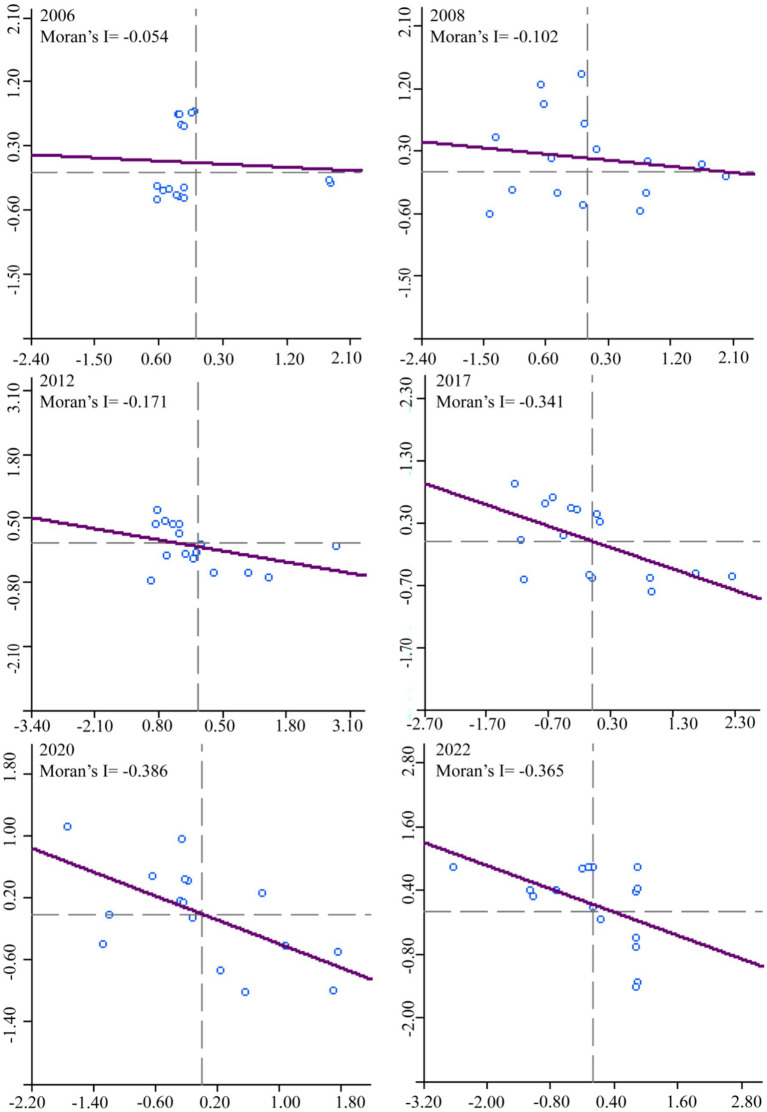
Moran’s I scatter plot during 2006–2022.

Local Moran’s I (Figure 5) demonstrates a consistent annual increase in the number of cities situated in the second and fourth quadrants, indicating persistent challenges in achieving coordinated UGLUE improvement across the Chengdu-Chongqing urban agglomeration.

#### 4.2.2 Hotspot analysis

**Figure 6:**
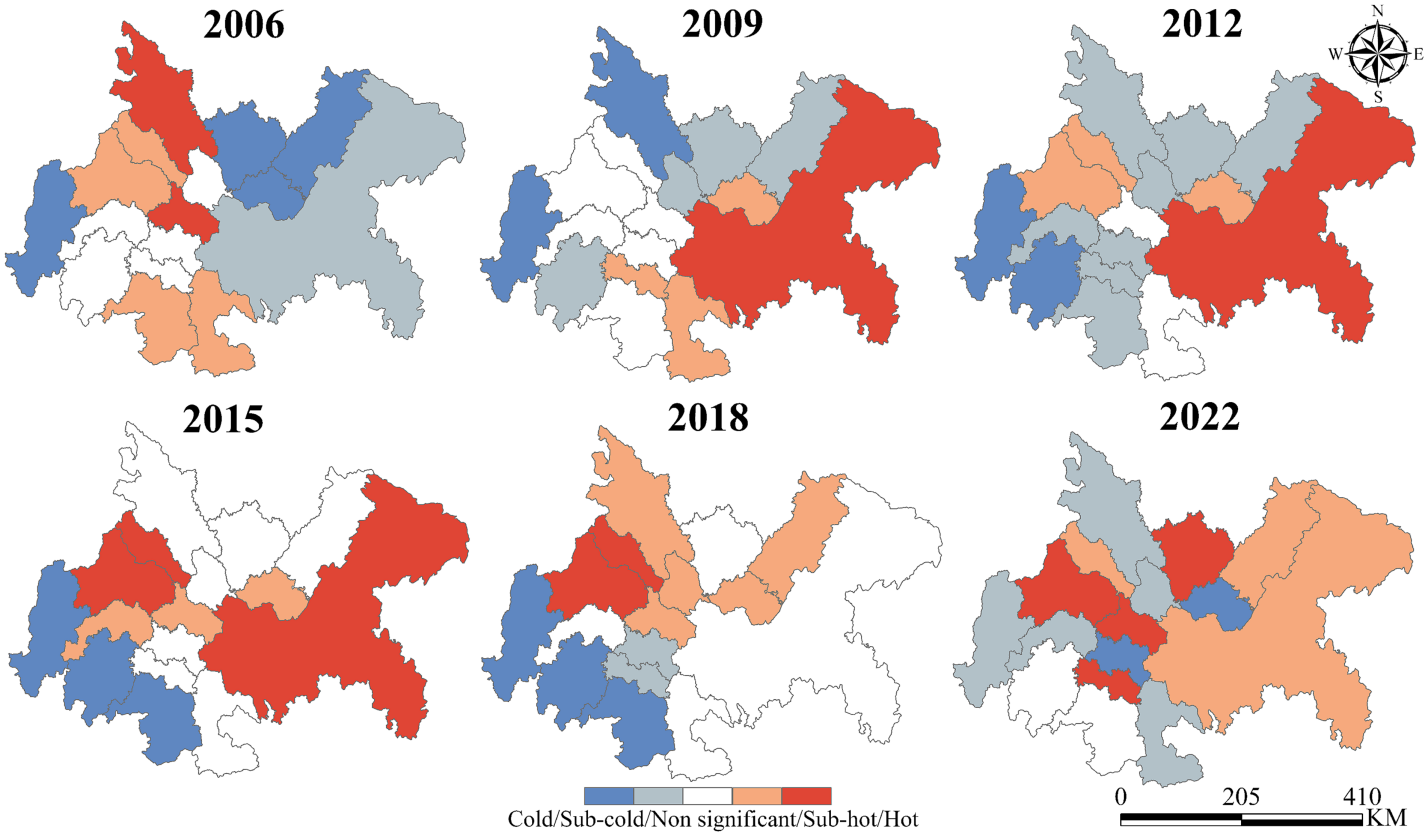
Spatial hotspot analysis of UGLUE in 2006–2022.

The spatiotemporal variations of hot and cold spots were mapped using ArcGIS Pro 3.0.2. Figure 6 illustrates the spatial hot spot analysis results. The number of cold spots and sub-cold spots increased from 5 in 2006 to 7 in 2022, demonstrating a clear expansion trend of cold zones. Cold spots have progressively expanded in peripheral cities such as Neijiang and Ya, forming a contiguous distribution by 2022, indicative of weak interregional cooperation.

#### 4.2.3 The standard deviation ellipse analysis

**Figure 7:**
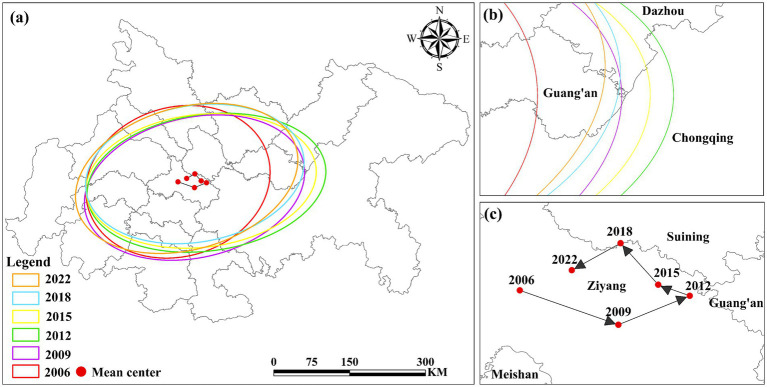
Standard deviation ellipse analysis of UGLUE in 2010–2022. **(a)** Evolutionary map of the UGLUE center of gravity for the Chengdu-Chongqing urban agglomeration; **(b)** for the trend of the enlarged center of gravity evolution; **(c)** represents the trajectory of the center of gravity.

The spatial–temporal evolution of UGLUE in the Chengdu-Chongqing urban agglomeration was analyzed using standard deviation ellipse methodology from 2006 to 2022. Figures 7a,b demonstrate that the UGLUE ellipse exhibits a predominant northeast-southwest orientation, reflecting the characteristic development pattern along the Chengdu-Chongqing axis. Figure 7c indicates that the mean center trajectory reveals distinct spatial dynamics: an eastward shift during 2006–2012, followed by a consistent westward movement in the subsequent decade (2012–2022).

#### 4.2.4 Identification result

The model results reveal significant obstacles to collaborative UGLUE improvement among cities in the Chengdu-Chongqing urban agglomeration from 2006 to 2022, with increasingly pronounced negative spillover effects indicating low regional cooperation levels. The urban agglomeration demonstrates limited capacity for fostering positive green cooperation through innovation diffusion, talent mobility, or regional integration. A particularly concerning spatial pattern emerges from hotspot analysis: the dual-core structure of Chengdu and Chongqing exhibits strong siphon effects, where their high UGLUE values paradoxically suppress the surrounding cities’ performance. This phenomenon stems from the agglomeration’s imbalanced development pattern, where the core cities dominate resource allocation, attracting labor forces and high-end talent while restricting factor mobility in peripheral areas.

Furthermore, the region suffers from disordered industrial gradient transfer. The substantial developmental gap between core and peripheral cities forces the latter to accept outdated, high-pollution industries from the core regions, as they lack the infrastructure to support more advanced sectors. This inefficient industrial transfer mechanism has exacerbated resource depletion and ecological degradation in surrounding areas, creating a self-reinforcing cycle of underdevelopment.

### 4.3 Analysis of the influence of urban expansion on UGLUE

#### 4.3.1 Baseline regression result

The baseline regression results defined in this study were obtained using STATA 16.0. Table 3 presents the estimated effects of UEI on UGLUE. The baseline regression in Column (1) reveals a significantly negative coefficient for UEI without control variables, suggesting an initial inverse relationship. Columns (2)–(6) progressively incorporate various control variables. Across all models, UEI consistently demonstrates statistically significant negative coefficients, reinforcing its adverse impact on UGLUE. These results indicate that UEI exerted a persistent and detrimental effect on UGLUE in the Chengdu-Chongqing urban agglomeration during 2006–2022, highlighting the challenges of balancing rapid land growth with sustainable land use.

**Table 3:** Baseline regression.

Variables	(1)	(2)	(3)	(4)	(5)	(6)
	Model 1	Model 2	Model 3	Model 4	Model 5	Model 6
*UEI*	−0.161^**^ (−2.26)	−0.173^**^ (−2.44)	−0.173^*^ (−2.09)	−0.175^**^ (−2.13)	−0.184^**^ (−2.26)	−0.185^**^ (−2.36)
*POP*		1.968^***^ (3.09)	1.460^**^ (2.34)	1.481^**^ (2.37)	1.563^**^ (2.21)	1.563^**^ (2.20)
*RE*			−0.005^**^ (−2.21)	−0.005^*^ (−2.09)	−0.005^**^ (−2.18)	−0.005^**^ (−2.18)
*TECH*				0.010 (0.23)	0.007 (0.17)	0.007 (0.17)
*IDS*					−0.101 (−0.81)	−0.100 (−0.81)
*GOV*						−0.024 (−0.13)
Constant	0.450^***^ (92.87)	−11.700^***^ (−2.98)	−8.103^*^ (−2.08)	−8.304^*^ (−2.10)	−8.717^*^ (−1.99)	−8.714^*^ (−1.98)
Year effect	Y	Y	Y	Y	Y	Y
City effect	Y	Y	Y	Y	Y	Y
Observations	272	272	272	272	272	272
R-squared	0.688	0.707	0.729	0.729	0.732	0.732

^***^p < 0.01, ^**^p < 0.05, ^*^p < 0.1.

#### 4.3.2 Robustness test

To verify the robustness of our findings, we implemented three validation approaches: (1) incorporating per capita GDP as an additional control variable, (2) truncating the distribution tails of the core dependent variable, and (3) substituting Urban Sprawl Index (USI) for UEI as the primary explanatory variable (Table 4). The regression coefficients for the core variables remained statistically significant and exhibited minimal variation across all robustness checks, confirming the reliability of our conclusions.

**Table 4:** Robustness test.

Variables	(1)	(2)	(3)
	Add control variables	Tail reduction treatment	Change the core explanatory variable
*UEI*	−0.172^**^	−0.185^**^	
*USI*			−0.014^**^
Constant	−11.596^**^	−8.720^*^	−7.399^*^
Control variable	Y	Y	Y
Year effect	Y	Y	Y
City effect	Y	Y	Y
Observations	272	272	272
R-squared	0.733	0.732	0.737

^***^p < 0.01, ^**^p < 0.05, ^*^p < 0.1.

This study employs an instrumental variable (IV) regression approach to investigate the causal impact of UEI on UGLUE, specifically addressing potential endogeneity issues. Drawing upon prior research (27), the number of fixed-line telephones (TEL) in 1984 and the number of POST stations in the Ming Dynasty (POST) are selected as the primary instrumental variables. These instruments are chosen for their capacity to reflect historical population density and economic activity, as well as their strong correlation with contemporary urban expansion, while satisfying the exogeneity requirement.

Analyses presented in (1) and (3) demonstrate a significant positive correlation between both TEL and POST and UEI across both instrumental variable specifications. Furthermore, the Kleibergen-Paap rk-LM and Cragg-Donald Wald F statistics consistently exceed the conventional threshold of 10, thereby mitigating concerns regarding weak instrumental variables and under-identification. This robust statistical evidence affirms the validity of the selected instruments.

When TEL is utilized as an instrumental variable, the estimated coefficient for UEI is −3.343 (*p* < 0.01), as reported in (2). Similarly, when POST serves as the instrumental variable, (4) reveals an estimated coefficient for UEI of −2.724 (*p* < 0.01). These findings consistently indicate a significant negative causal relationship between UEI and UGLUE, thereby substantiating the robustness of this study’s conclusions (Table 5).

**Table 5:** Instrumental variable method.

Variables	TEL results		POST results	
	(1)	(2)	(3)	(4)
	UEI	UGLUE	UEI	UGLUE
*UEI*		−3.343^***^ (1.095)		−2.724^***^ (1.029)
*TEL*	0.145^***^ (0.034)			
*POST*			0.158^***^ (0.037)	
Kleibergen-Paap rk LM	18.25^***^		17.57^***^	
Cragg-Donald Wald F	17.98		17.46	
N	273	273	273	273

^***^p < 0.01, ^**^p < 0.05, ^*^p < 0.1.

#### 4.3.3 Heterogeneity test

This study further categorized the samples into eight groups based on their characteristics: transportation hub cities (TH), key environmental protection cities (KE), central cities (CN), and resource-based cities (RB). The results are presented in Table 6. With the exception of central cities, the influence coefficients of UEI on UGLUE were negative across the remaining categories, and these coefficients generally passed the significance test. This finding corroborates the study’s conclusion that “UEI tends to impede the enhancement of UGLUE.” More specifically, UEI in transportation hub cities, key environmental protection cities, non-central cities, and resource-based cities exhibits a more pronounced negative impact on UGLUE, with transportation hub cities demonstrating the strongest negative effect.

**Table 6:** Heterogeneity test.

Variables	(1)	(2)	(3)	(4)	(5)	(6)	(7)	(8)
	TH	N-TH	KE	N-KE	CN	N-CN	RB	N-RB
*UEI*	−0.611^***^	−0.179^**^	−0.210^*^	−0.039	0.226^*^	−0.117	−0.145^*^	−0.107
Control	Y	Y	Y	Y	Y	Y	Y	Y
Year effect	Y	Y	Y	Y	Y	Y	Y	Y
City effect	Y	Y	Y	Y	Y	Y	Y	Y
Observations	34	238	170	102	68	204	119	153
R-squared	0.935	0.710	0.805	0.720	0.856	0.712	0.695	0.775

^***^p < 0.01, ^**^p < 0.05, ^*^p < 0.1.

#### 4.3.4 GTWR

To address the inherent limitation of Ordinary Least Squares (OLS) in fully revealing complex spatial–temporal heterogeneity, this study rigorously compared the model values of various spatial regression methods, including Geographically and Temporally Weighted Regression (GTWR), Geographical Weighted Regression (GWR), and Multiscale Geographically Weighted Regression (MGWR). As shown in Table 7, the results unequivocally indicate that GTWR is the most reliable model, offering superior explanatory power and accuracy for capturing dynamic spatiotemporal interactions.

**Table 7:** Model values of GTWR, GWR and OLS.

Model	GTWR	GWR	MGWR
Bandwidth	0.174	0.193	/
Residual Squares	7.182	10.421	15.532
Sigma	0.183	0.214	1.055
AICc	10.158	34.745	53.050
R2	0.852	0.614	0.029
R2Adjusted	0.849	0.608	0.024

**Figure 8:**
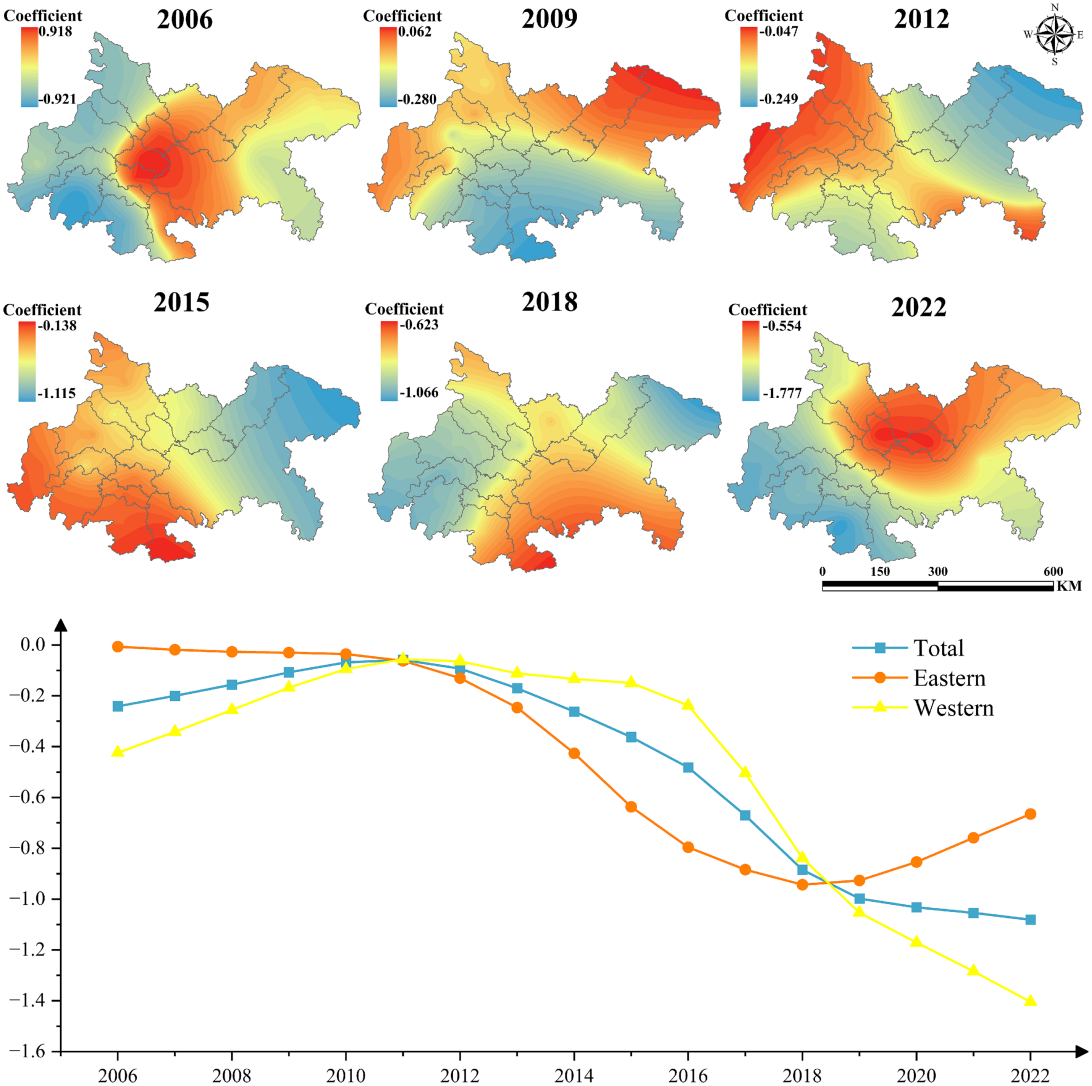
The impact of UEI on UGLUE (2006–2022).

As illustrated in Figure 8, the average influence coefficient of UEI on UGLUE exhibits a significant linear decline, decreasing from −0.24 in 2006 to −1.08 in 2022. This trend indicates that, as urbanization accelerates, urban expansion in the Chengdu-Chongqing urban agglomeration exerts an increasingly adverse effect on land-use environmental efficiency. The findings corroborate the prevailing development pattern in this region, wherein economic growth is prioritized at the expense of ecological sustainability.

Spatial heterogeneity analysis further reveals that, except during 2011–2018—when the eastern sub-region exhibited a weaker negative impact than the western sub-region—the western urban agglomeration generally experienced less pronounced adverse effects. Moreover, Chengdu and Chongqing, as the core cities of the agglomeration, consistently demonstrated a less detrimental influence. This observation suggests that factor concentration in central cities fosters more advanced industrial structures and infrastructure, thereby enhancing environmental governance efficiency through economies of scale.

**Figure 9:**
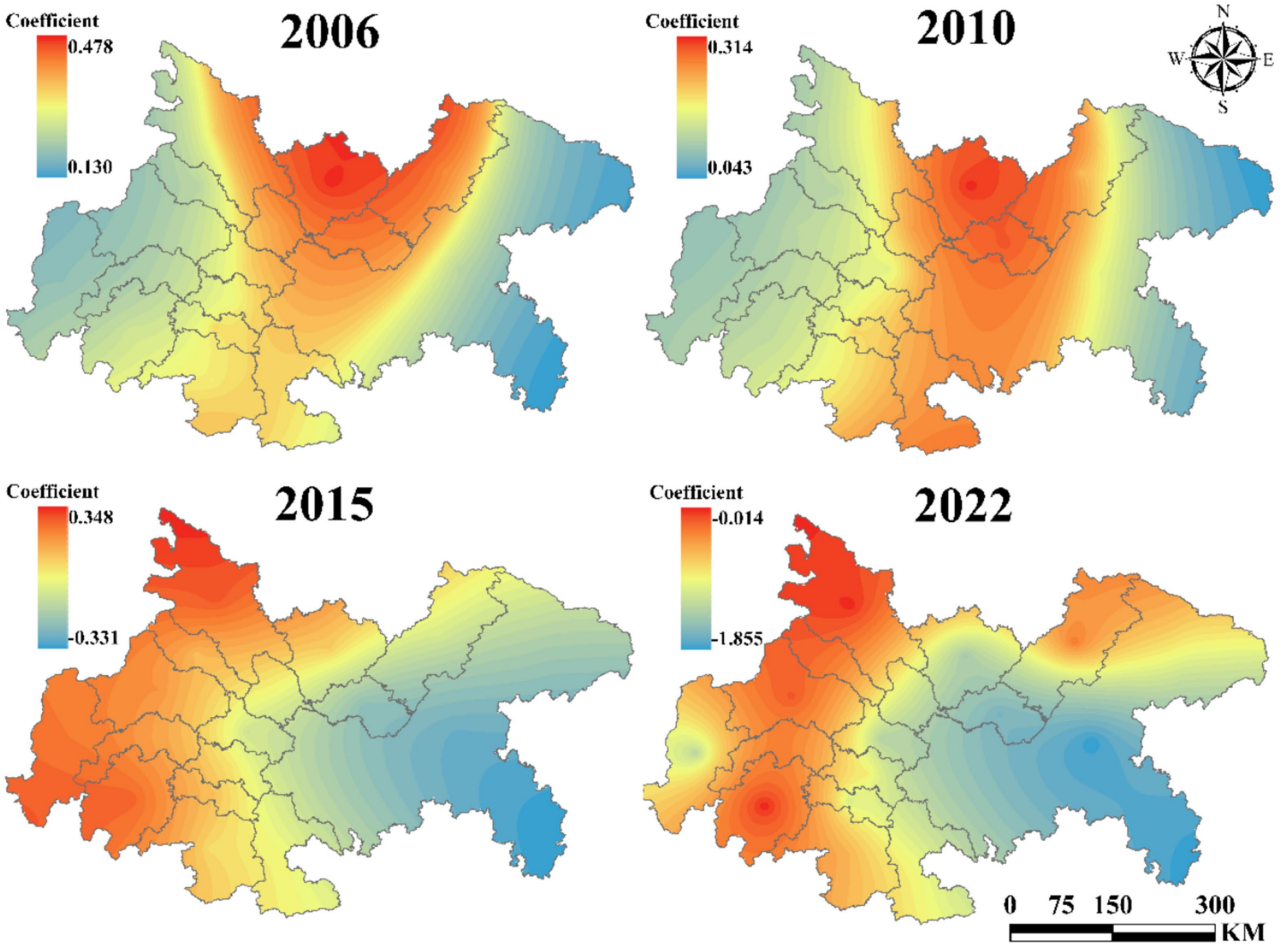
The impact of POP on UGLUE (2006–2022).

Figure 9 illustrates the influence coefficient of population density on UGLUE. From 2006 to 2010, the coefficient remained positive across all regions, suggesting that population concentration facilitated the efficient flow of economic and production factors, thereby enhancing land-use efficiency. However, by 2015, the coefficient turned negative in Chongqing, and by 2022, it became negative throughout the entire region. This shift indicates that the adverse effects of population agglomeration—such as resource inefficiency and environmental degradation—have begun to outweigh its benefits, ultimately impeding UGLUE.

**Figure 10:**
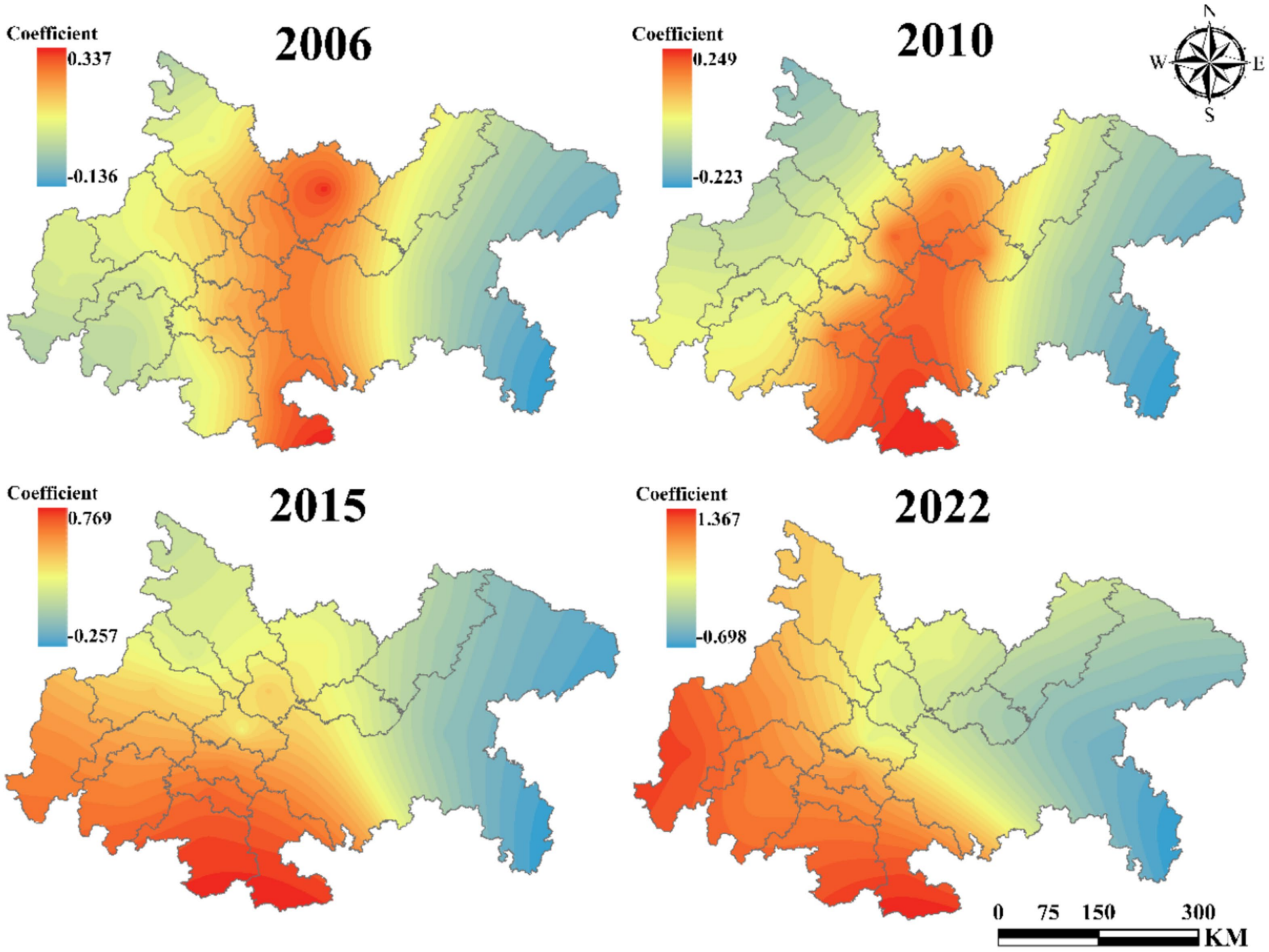
The impact of TECH on UGLUE (2006–2022).

**Figure 11:**
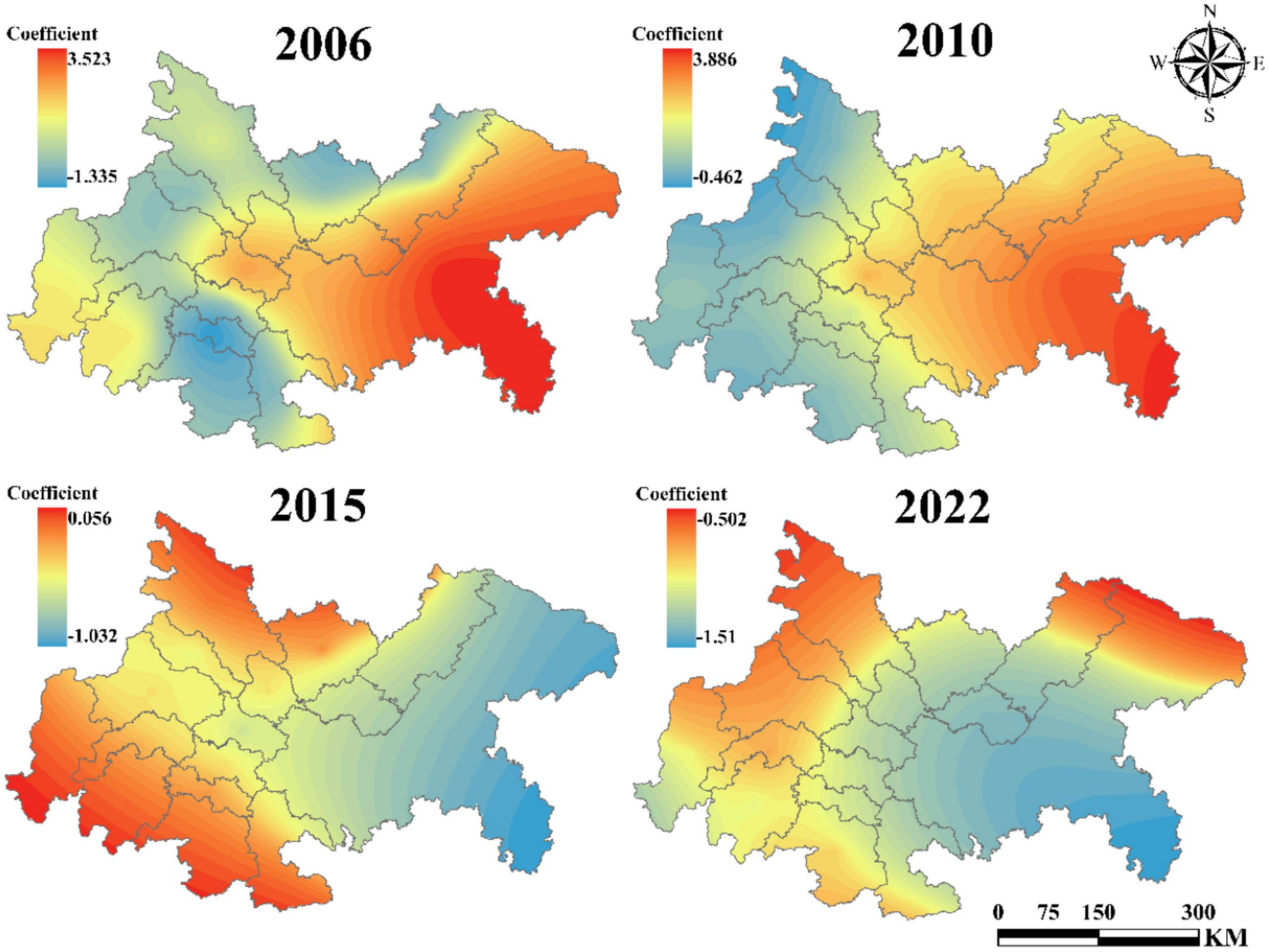
The impact of GOV on UGLUE (2006–2022).

**Figure 12:**
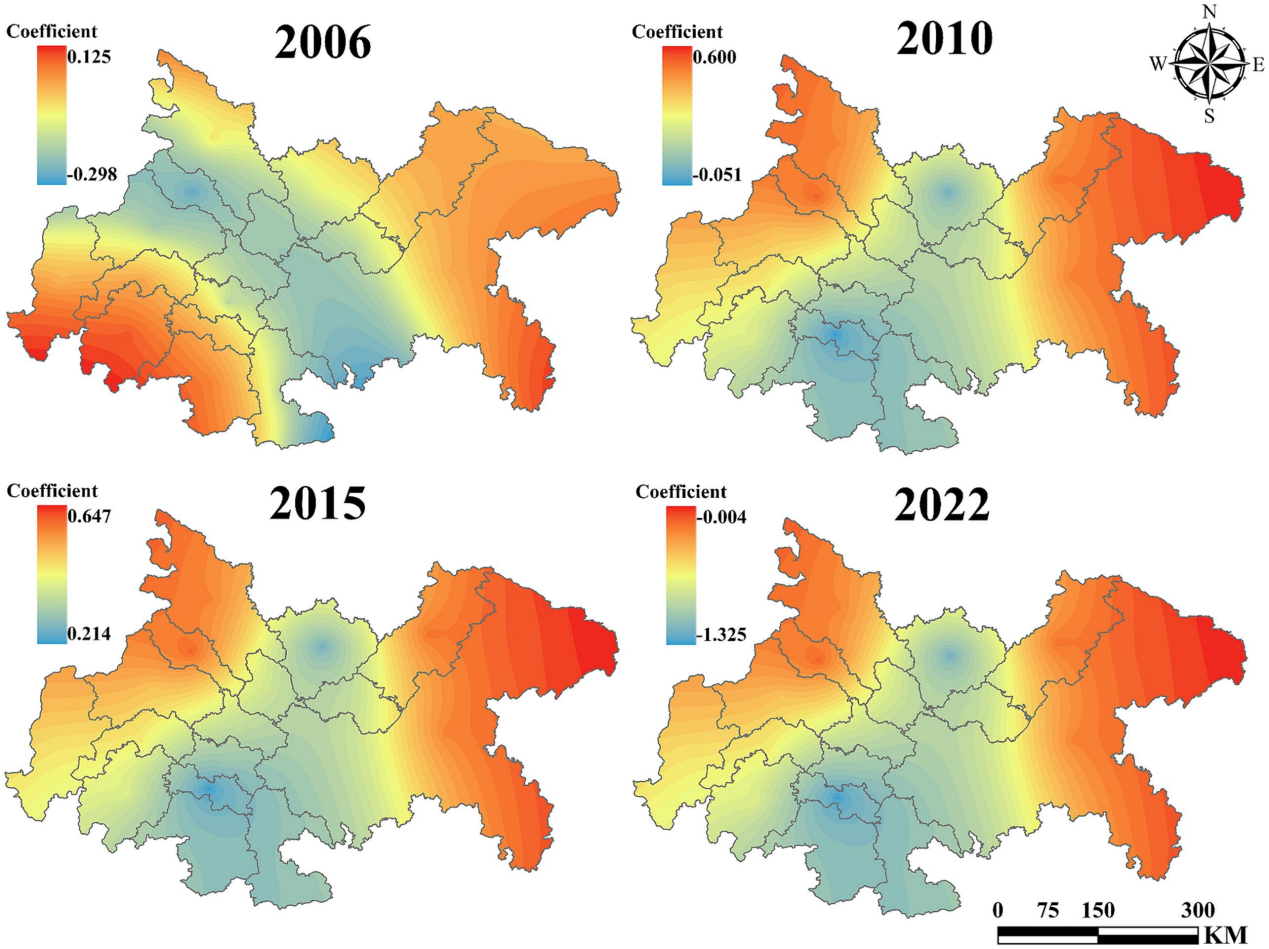
The impact of IDS on UGLUE (2006–2022).

**Figure 13:**
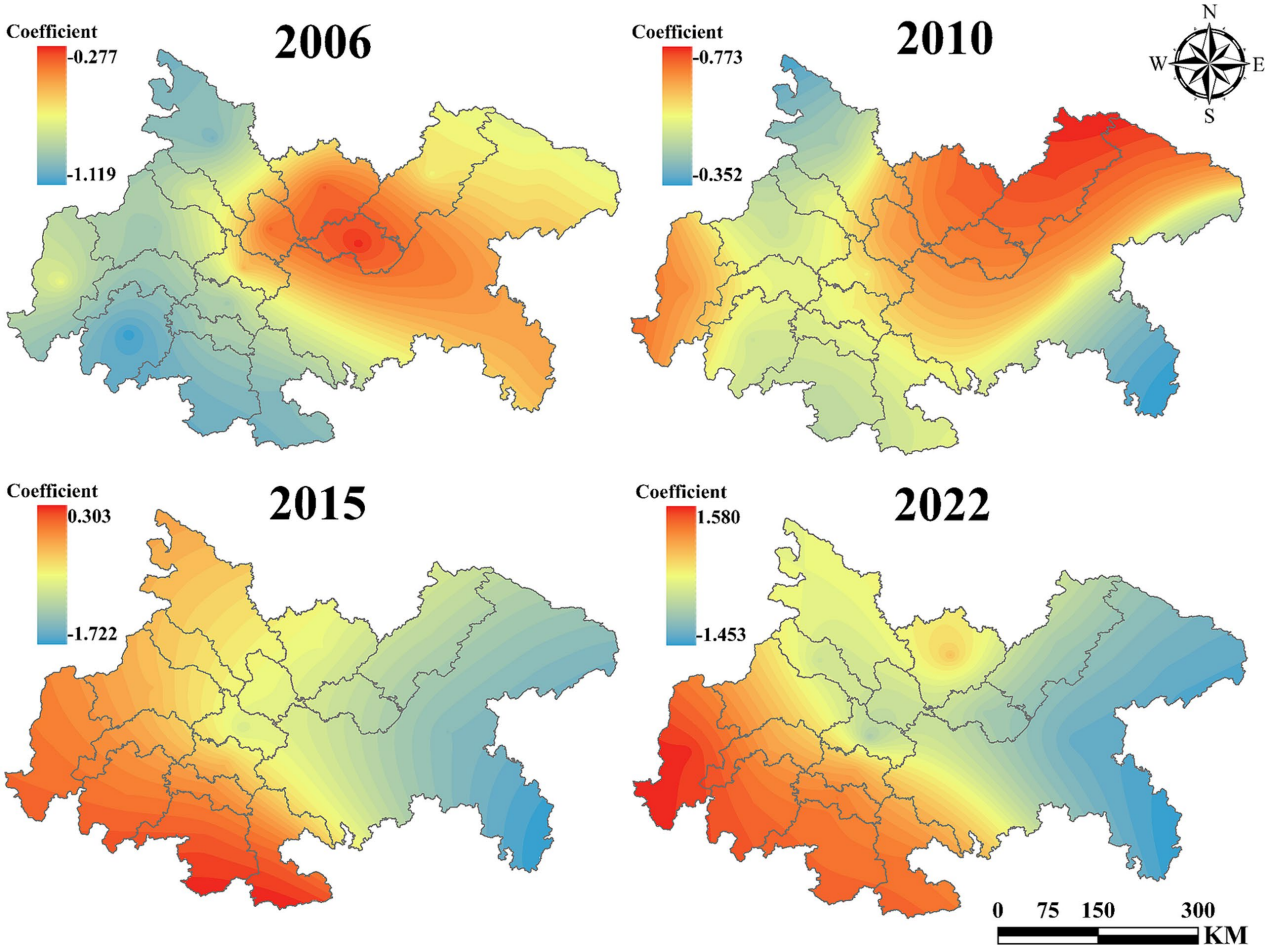
The impact of RE on UGLUE (2006–2022).

Figure 10 presents the influence coefficient of technological level on UGLUE. The results demonstrate consistently positive coefficients for cities in the central and western regions of the Chengdu-Chongqing urban agglomeration, with a strengthening trend over time. Conversely, the negative impact in eastern cities has shown significant expansion. Figure 11 reveals the effect of government intervention. During 2006–2010, the eastern region exhibited strongly positive coefficients that increased annually. However, after 2015, these coefficients turned negative, while central and western regions maintained relatively stable impacts. Figure 12 displays the impact of industrial structure upgrading on UGLUE. This factor shows remarkable stability, with consistently positive coefficients in eastern and western urban agglomerations. The central region maintained negative coefficients throughout the study period, except for 2015. Figure 13 illustrates the spatial–temporal evolution of resource efficiency’s influence on UGLUE. The entire region showed negative coefficients in 2006 and 2010. However, by 2015–2022, central and western regions began demonstrating positive influences. This pattern suggests that cities surrounding Chengdu effectively balance economic and ecological efficiency, where improved resource efficiency contributes to steady UGLUE enhancement.

## 5 Discussion

### 5.1 The UGLUE has been steadily improving, demonstrating a positive optimization trend; however, significant internal disparities still persist

Within China’s new urbanization context, pursuing “smart growth” strategies and enhancing the green, efficient utilization of land resources have become crucial for achieving sustainable development objectives. This study investigates the spatial–temporal impacts of urban expansion on green land use efficiency (UGLUE) in the Chengdu-Chongqing urban agglomeration, providing valuable theoretical and empirical insights for balancing urban development with ecological sustainability. The results demonstrate significant dynamic optimization in UGLUE across the study region, with average values showing marked improvement from 2006 to 2022. This upward trend mirrors findings by Tan et al. (17) in the Yangtze River Delta during 2004–2015 and reflects broader national developments, including the greening of industrial structures, improvements in ecological compensation mechanisms, and expansion of infrastructure networks (52). The spatial configuration of UGLUE has evolved from a “dual-core polarization” pattern to a more balanced “multi-center collaboration” system, consistent with Zhou et al.’s (40) observations of narrowing regional disparities after 2015. The UGLUE measurement results in this study are consistent with the broader national trend of increasing UGLUE across most regions of China. This upward trajectory is largely attributable to the deepening implementation of green development strategies, the continuous optimization of urban land-use structures, and increased investment in environmental governance—all of which have significantly enhanced the allocation efficiency of green land resources and their associated ecosystem services (40, 53).

However, this study further reveals that limitations in factor mobility and institutional shortcomings have constrained improvement in certain peripheral cities such as Yaan and Meishan, where UGLUE remains relatively stagnant. This reflects a pronounced spatial gradient within the Chengdu–Chongqing urban agglomeration and underscores the persistence of spatial inequality in land-use efficiency. These findings highlight the urgent need for targeted policy interventions to address structural bottlenecks in underperforming areas. Ultimately, this research contributes to ongoing discussions on sustainable urban expansion in emerging economies, particularly regarding the spatiotemporal dynamics of land-use efficiency under rapid urbanization.

### 5.2 The UGLUE exhibits a significant spatial negative autocorrelation effect

Using Exploratory Spatial Data Analysis (ESDA) and standard deviation ellipse techniques, this study investigates the spatiotemporal evolution of UGLUE in the Chengdu–Chongqing urban agglomeration from 2006 to 2022, effectively addressing the scientific question of spatial spillover effects of UGLUE in the context of regional development imbalance. The global Moran’s I analysis reveals persistent negative spatial spillover effects throughout the study period, with increasing local Moran’s I (H-L quadrant) values further confirming the absence of positive spillover transitions.

These findings stand in sharp contrast to existing studies on urban land use efficiency. For instance, Wu et al. (54) reported a significantly positive global Moran’s I for land use efficiency in the Yellow River Basin, with spatial spillover effects strengthening over time. Similarly, Zhang et al. (60) revealed that, at the provincial level, China’s sustainable land use efficiency exhibited a consistently positive spatial spillover effect from 2000 to 2020, with increasingly pronounced spatial agglomeration patterns. Chen et al. (61) also found that cities in the Yangtze River Basin experienced growing positive spatial spillovers in land use efficiency year by year. Xue et al. (55) further demonstrated a significantly positive global Moran’s I for land use efficiency in the Guanzhong Plain urban agglomeration. All of the aforementioned studies, conducted in different urban contexts across China, consistently reported positive spatial autocorrelation patterns. The observed spatial disparities stem from the region’s distinctive dual-core development structure. Chengdu and Chongqing exhibit pronounced resource siphon effects that constrain coordinated regional development, differing fundamentally from more economically integrated urban agglomerations like the Yangtze River Delta or Pearl River Delta regions. The latter benefits from established industrial complementarity and positive spillover effects, whereas the Chengdu-Chongqing region demonstrates incomplete industrial gradient transfer. Peripheral cities lack the necessary infrastructure and development capacity to effectively absorb industrial transfers from core cities, resulting in weak industrial coordination and constrained green development potential. These findings highlight the complex challenges facing emerging dual-core urban agglomerations in achieving sustainable land use efficiency. The study underscores the critical need for integrated policy interventions that simultaneously address regional development imbalances, enhance industrial coordination mechanisms, improve ecological compensation systems, and strengthen inter-city cooperation frameworks. Such multidimensional approaches are essential for promoting sustainable urbanization patterns in developing regions with similar spatial-economic configurations.

### 5.3 The impact of urban expansion on UGLUE in the Chengdu–Chongqing urban agglomeration is not bidirectional in causality and operates through a complex, nonlinear mechanism

The findings reveal that the impact of urban expansion on UGLUE in the Chengdu–Chongqing urban agglomeration is characterized by pronounced nonlinearity and spatiotemporal heterogeneity. This study further identifies the causal relationship between urban expansion and UGLUE and confirms the absence of bidirectional causality, thereby addressing one of the central scientific questions posed. While earlier studies (4) suggested that urban expansion could enhance UGLUE through industrial upgrading and increased economic vitality, our results offer a more nuanced perspective.

The application of the Geographically and Temporally Weighted Regression (GTWR) model effectively overcomes the limitations of conventional econometric methods in capturing spatial heterogeneity. The results derived from GTWR not only fulfill the study’s objective of identifying spatially heterogeneous effects but also provide more precise spatial coordinates to inform targeted policymaking. By 2022, urban expansion began to exert a negative influence on UGLUE at the regional scale, corroborating the conclusions of Zhou et al. (40). Although initial phases of urban expansion contributed to resource agglomeration and efficiency gains, the temporal analysis reveals a transition wherein emerging urban challenges—such as traffic congestion, environmental degradation, and spatial imbalance (56–59)—have undermined earlier gains.

These results suggest that the region’s previous growth model, centered on rapid expansion over quality development, has reached a critical inflection point. Uncontrolled urbanization is now eroding the prospects for sustainable land use. The findings highlight the urgent need for a paradigm shift in urban planning and development strategy, pointing toward three key policy directions: First, the implementation of tailored urban expansion strategies aligned with the region’s development stage can help reconcile growth objectives with environmental constraints. Second, enhancing regional industrial specialization and strengthening inter-city cooperation can improve resource allocation efficiency and mitigate negative spillover effects. Third, strict enforcement of ecological redlines and resource utilization caps is essential to safeguard against unsustainable development patterns.

These findings carry broader implications for emerging urban agglomerations worldwide, particularly those undergoing similar rapid urbanization trajectories. The study underscores the necessity for adaptive, spatially sensitive governance approaches, as the benefits of early-stage expansion inevitably give way to more complex sustainability challenges.

### 5.4 Limitations and future research directions

This study acknowledges several inherent limitations in its scope and data coverage. Due to data availability constraints, only a few core cities, such as Chengdu and Chongqing, have updated records extending to 2023, while most other cities only provide economic, social, and urban construction data up to 2022. To ensure data consistency across the full sample, the study period was limited to 2006–2022. This may slightly restrict the timeliness of the findings and limit the study’s ability to capture the impacts of the most recent policy interventions or urban development trends. In addition, key indicators directly related to the built environment—such as building volume and height—were not incorporated into the UGLUE evaluation system due to data unavailability, which may affect the comprehensiveness of the assessment.

The generalizability of the results also warrants cautious interpretation. Although the Chengdu–Chongqing urban agglomeration serves as a representative case of rapid urbanization in emerging economies, its unique dual-core development pattern and region-specific policy framework may constrain the applicability of the findings to urban agglomerations with different spatial-economic structures or stages of development. Nevertheless, the spatial structure of “dual-core drive and multi-polar support” represented by the Chengdu–Chongqing case offers a valuable reference for other late-developing urban regions worldwide. The model of strategic industrial clustering and regional integration it demonstrates is particularly instructive for areas striving to balance rapid urban expansion with sustainable land use and green transformation.

Future research should aim to extend the study window by incorporating more recent data and integrating additional indicators—such as building volume, height, and urban form complexity—to improve the robustness and comprehensiveness of the UGLUE evaluation framework. Comparative studies across multiple urban agglomerations, both within and outside of China, would further enhance the generalizability of the findings and contribute to a broader understanding of the relationship between urban expansion and green land use efficiency under varying institutional and spatial development contexts.

## 6 Conclusions and policy implications

This study takes the Chengdu–Chongqing urban agglomeration as a representative case to systematically examine the impact mechanism of urban expansion on UGLUE, with particular attention to its spatial spillover effects and spatiotemporal heterogeneity. By integrating ESDA, standard deviation ellipse analysis, fixed-effects models, instrumental variable (IV) regression, and Geographically and Temporally Weighted Regression (GTWR), the research addresses three core scientific questions: whether urban expansion weakens UGLUE, whether the mechanism is stable, and whether regional development imbalances generate spatial feedback effects.

First, UGLUE across the Chengdu–Chongqing urban agglomeration showed a significant upward trend from 2006 to 2022, indicating that green development strategies and policy interventions have produced tangible effects at the regional level. However, substantial spatial gradients and temporal disparities remain within the urban cluster. Chengdu and Chongqing, as twin core cities, have consistently outperformed, while peripheral cities such as Ya’an, Meishan, and Neijiang have shown only modest improvements. This pattern highlights a strong correlation between green land allocation efficiency and factors such as industrial base, policy intensity, and administrative capacity.

Second, the study identifies a pronounced spatial negative spillover effect of UGLUE using spatial econometric techniques, wherein high-efficiency cities have not successfully driven coordinated improvement among their neighbors. Unlike other regions in China that commonly exhibit positive spatial spillovers, the Chengdu–Chongqing urban agglomeration suffers from a “dual-core siphoning—peripheral lag” dynamic, wherein resources, technologies, and green industries remain concentrated in core cities, reinforcing regional inequality and exposing the institutional limits of ecological coordination.

Third, the empirical analysis reveals a significant negative impact of urban expansion on UGLUE, and importantly, no evidence of bidirectional causality. Urban expansion, characterized by inefficient land use, fragmented functionality, and ecological encroachment, undermines green performance, particularly in transport hubs and resource-based cities. GTWR results further illustrate the temporal and spatial variation of this relationship, confirming a nonlinear evolution pattern: initial expansion may promote efficiency, but prolonged sprawl reverses those gains. The southwestern subregion was found to be especially vulnerable to negative effects, underscoring the urgency of implementing rigid ecological boundaries and growth boundary management.

Finally, this study contributes methodological innovation by constructing a multi-scale framework for identifying spatial heterogeneity and offers theoretical value by proposing a causal chain of “expansion intensity → efficiency decline → negative spillover.” This framework enhances our understanding of how land-use efficiency evolves under rapid urbanization. From a policy perspective, the study highlights three strategic directions: Establish a joint regional coordination platform led by Chengdu and Chongqing to foster resource sharing and integrated spatial planning; Strengthen targeted support for peripheral cities through land-use regulation, infrastructure investment, and industrial upgrading; Promote a quality-oriented urban expansion model that prioritizes compact city development and brownfield regeneration to minimize ecological degradation.

In conclusion, this study not only advances the empirical understanding of the causal linkages and spatial externalities between urban expansion and UGLUE but also provides actionable policy insights for China and other rapidly urbanizing regions worldwide pursuing sustainable land-use transformation.

## Data Availability

The data analyzed in this study is subject to the following licenses/restrictions: data sets are available on reasonable request from the corresponding author. Requests to access these datasets should be directed to CW, wangc6292@126.com.
